# Evaluation of Multidimensional Functional Impairment in Adult Sexual Assault Survivors, with a Focus on Its Psychological, Physical, and Social Dimensions, Based on Validated Measurements: A PRISMA Systematic Review

**DOI:** 10.3390/ijerph20146373

**Published:** 2023-07-15

**Authors:** Thibault Schalk, Juliette Oliero, Emma Fedele, Victor Trousset, Thomas Lefèvre

**Affiliations:** 1 AP-HP, Jean Verdier Hospital, Department of Legal and Social Medicine, Avenue du 14 Juillet, F-93140 Bondy, France; 2 UFR SMBH, Sorbonne Paris Nord University, UFR SMBH, F-93100 Bobigny, France; 3 IRIS—Institut de Recherche Interdisciplinaire Sur les Enjeux Sociaux, UMR CNRS 8156 Inserm 997 EHESS USPN, Campus Condorcet, 5 Cour des Humanités, F-93300 Aubervilliers, France

**Keywords:** sexual assault, functional outcomes, quality of life

## Abstract

Sexual violence (SV) is widely prevalent around the world: according to studies, 18 to 51% of women and 1 to 9% of men experience it at some point in their lives. Yet, experiences of SV are rarely disclosed outside the private sphere. Pathologies, acute or chronic, can be associated with SV. The study of the links between SV and health is often fragmented, viewed through the lens of a specific pathology, yet SV certainly has an impact on the different dimensions of the functioning of survivors (physical, psychological, social, and so on), whether or not there is an identified pathology at the origin of this impact. No synthesis of the knowledge on functional impairment in adult sexual assault survivors has been identified to date. Therefore, we conducted a systematic review according to the PRISMA recommendations, focusing on the assessment via validated scales or standardized measurements of the different dimensions of functional impairment in sexual assault survivors aged 15 and over, excluding abuse in childhood and polyvictimization. We searched the Medline database from its inception to October 2022, identifying 1130 articles. Two evaluators carried out their analysis, and fifty-one articles were retained. In the end, only 13 articles were included. Their quality was assessed by referring to their compliance with STROBE recommendations. Of these 13 articles, only 4 have a quality level deemed to be satisfactory, and they relate to 4 dimensions of functioning: psychological, sexual, physical (pain), and social. The main results were that survivors reported increased restrictions of activities, sexual dysfunctions such as vulvodynia or dyspareunia, decreased social satisfaction and functioning, and decreased self-esteem and quality of life compared to the general population. To date, evidence for functional impairment is very limited, preventing researchers and clinicians from gaining clear and well-established knowledge about the functioning of sexual assault survivors. Research in this area needs to evolve urgently.

## 1. Introduction

The World Health Organization defines sexual violence as “any sexual act, attempt to obtain a sexual act, or other act directed against a person’s sexuality using coercion, by any person regardless of their relationship to the victim, in any setting” [1].

Its prevalence throughout the world is significant, though it varies across countries and studies. For example, one in five women in the United States have suffered sexual violence according to the Center for Disease Control and Prevention (CDC) [2]. Another study [3], published in 2014, found that 43.9% of American women and 23.4% of American men were survivors of sexual violence and that 19.3% of American women and 1.7% of American men had been raped at some point in their lives. A 2019 study [4] shows that 6.5% of American women (3 million) reported forced sexual experiences during their first sexual encounter. Other studies [5,6,7,8] around the world describe the lifetime prevalence of sexual assault as ranging from 18 to 51% for women and 1 to 9% for men.

The scientific literature shows or suggests associations between sexual violence and multiple pathological consequences, somatic [9,10] or psychological [11,12,13,14], acute or chronic. Some of these pathologies precede or are consecutive to sexual violence. Knowing the diversity of pathologies associated with sexual violence is important, in particular, because they can sometimes be the reason for consultation and recourse to care, which may cause the violence to be disclosed to a health professional. However, this has three important limits. Firstly, it means reducing violence and people to their pathological consequences, whereas health is more than pathology or the absence of pathology, and knowing the impact of an event on a person’s life is also important in order to better guide the orientation of these people [15]. Secondly, knowing the association between violence and a pathology says nothing specific about the severity of the impairment and the degree of functional impact, even if it suggests that there may be an impact associated with the pathology. For example, the intrusive symptoms of post-traumatic stress disorder (PTSD) cause a priori [16] a more or less significant functional impairment. Thirdly, there may exist a priori a functional impairment without there being a specific pathology involved, or the pathologies may also combine, potentially reinforcing their impact on functioning.

There is, therefore, a lack of knowledge around functional impairment, in its different dimensions (psychological, physical, social, professional, etc.), knowledge that would allow us to take better account of what adult sexual assault survivors experience and which would be complementary to the knowledge and the appropriate care of the different associated pathologies. The knowledge established in this field has not to date been the subject of a synthesis.

The aim of this study is thus twofold: to identify the direct, standardized evaluations of functional impairment in adult sexual assault survivors in different dimensions, and to highlight, where appropriate, how this research remains underinvested with regard to the importance of the problem.

For this review, we focused on adults reporting isolated sexual violence, for which one or more dimensions of functional impairment were assessed by validated measures or scales. We did not consider the evaluation of functional impairment in child or adult survivors of childhood sexual violence, nor functional impairment linked to polyvictimization, i.e., to a combination of types of violence (sexual, domestic, etc.). Firstly, either because the population of minors is specific and is not evaluated in the same way as that of adults, or because it is likely that the nature or the intensity, or simply the mechanisms leading to a functional impact in adulthood linked to sexual violence in childhood, are different from those for violence occurring in adulthood, and secondly, because we initially wanted to isolate the two main elements of a type of (sexual) violence and functional impairment, so that the association between the two is the most plausible, without its interpretation being complicated by the accumulation of violence of various kinds.

## 2. Materials and Methods

### 2.1. Search Strategy

To carry out an inventory of studies on the direct evaluation by validated measures of functional impairment in adult sexual assault survivors, in its different dimensions, we conducted a systematic review according to the PRISMA recommendations [17].

We used the Medline database, and we considered all articles from its inception to 31 October 2022.

The research equation is a translation of the three concepts used: sexual violence (according to the WHO definition); the use of validated measures or scales; and the evaluation of functional impairment in several dimensions. The dimensions considered are the following: psychological, physical, social, professional, sexual impact, and quality of life.

We used the following equation:

“Quality of Life”[Mesh] OR “Activities of Daily Living”[Mesh] OR “quality of life” OR “ Activities of daily living” OR “Physical Fitness”[Mesh] OR “Sexual Dysfunctions, Psychological”[Mesh] OR “Sexual Health”[Mesh] OR “Occupational Health”[Mesh] OR “Pain”[Mesh] OR « Physical function* » OR «Physical impairment» OR «Physical dysfunction» OR «Physical failure» OR «Physical consequences» OR «Physical repercussions» OR «health consequences» OR “Physical disturbance” OR “functional impairment” OR “functional impairment” OR “sexual function*) OR «Psychological function*» OR «Psychological impairment» OR «Psychological dysfunction» OR «Psychological repercussions» OR habits OR «social dysfunction» OR «social impact» OR «life-changing» OR “social support” OR “social interaction” OR “social impairment” OR “social disturbance” AND assessment OR scale OR “evaluation scale” OR index OR questionnaire AND “Sex Offenses”[Majr:NoExp] OR “Rape”[Mesh] OR “Crime “[Mesh:NoExp]) OR Rape OR «sexual violence» OR «sexual assault» OR «sexual abuse» OR «sexual victimization» OR “sexual harassment” NOT “child abuse” OR child OR “child abuse” NOT “Adult Survivors of Child Abuse”[Mesh] OR “Child Abuse, Sexual”[Mesh]. The terms corresponding to the exclusion criteria are the following: NOT “child abuse” OR child OR “child abuse” NOT “Adult Survivors of Child Abuse”[Mesh] OR “Child Abuse, Sexual”[Mesh].

### 2.2. Inclusion and Exclusion Criteria

Inclusion criteria: studies on the direct measurement of one or more dimensions of functional impairment in adult sexual assault survivors; by a validated measure or scale; in people aged 15 and over. The forms of sexual violence considered included rape, sexual assault, sexual harassment, sexual abuse, or sexual contact. This violence had to have been experienced from the age of 15 years or older. Studies had to be peer-reviewed articles.

Exclusion criteria: studies on a pediatric population (under 15 years); systematic reviews and meta-analyses (if they existed); studies with fewer than 10 people; qualitative studies; studies on polyvictimization.

Two evaluators (TS and JO) blinded to each other screened all the articles identified by the search equation and applied the inclusion and exclusion criteria. At the end of this process, if there was disagreement between the two reviewers about whether or not to include a study in the review, a third reviewer (TL) analyzed the study and decided whether or not to include it.

The first step consisted of the two evaluators screening the articles on the basis of their title and abstract. Then, from this initial selection, a final selection was made based on a full reading of the studies retained in the first stage.

### 2.3. Analysis Strategy

To analyze the included articles, we created a table containing the authors of the articles and the journal of publication, the main objective of the study, the population studied, the type of sexual assault studied, the domain and function studied, the validated rating scales with Cronbach’s alpha coefficient if available or applicable, the statistical analysis used, and the results. The Cronbach coefficient is a statistic used to measure the internal reliability of a scale. Its value ranges from 0 to 1 and is considered acceptable if it is greater than 0.7 [18].

### 2.4. Quality Assessment of the Included Studies

As the included studies were all observational studies, we assessed their quality based on their level of compliance with the STROBE recommendations [19].

## 3. Results

### 3.1. Selection of Studies

**Figure 1:**
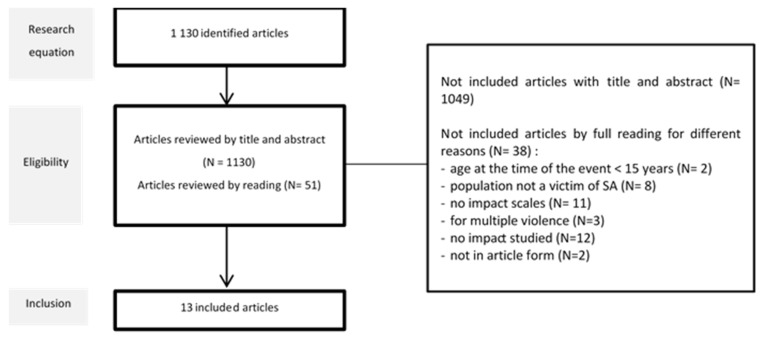
Flow chart of inclusion.

The search equation retrieved 1130 articles indexed in Medline from its inception to 31 October 2022, of which 51 were selected for further screening (Figure 1). Most excluded studies focused on a single pathology, such as PTSD or depression, or described factors of pathology. From the 51 articles selected, 38 were removed: 2 because the participants were under 15 at the time of the assault; 8 because the study populations were not sexual assault survivors; 11 because they did not use validated scales; 3 because they dealt with polyvictimization; 12 because they did not describe functional impairment; and 2 because they were book chapters.

### 3.2. Characteristics of These Articles

#### 3.2.1. General Characteristics of the Included Articles

Our review includes six longitudinal observational studies, five cross-sectional observational studies, and two case-control studies. Six articles have control groups. Seven studies recruited patients through an emergency department or casualty center, while the others recruited via questionnaire or telephone call. On average, the sample size in these studies is 230 people (with a minimum of 35 and a maximum of 935). The results are reported in Table 1.

These 13 studies dealt with the following dimensions of functional impairment: physical, sexual, psychological, and social functioning, as well as quality of life.

#### 3.2.2. Physical Impairment

Two studies [22,25] report increased physical pain and increased symptoms, such as difficulty concentrating, taking more time to think, and discomfort with light or noise in everyday life after sexual assault. These pains and symptoms increase within six weeks after the assault. There is a significant relationship between the body area that was painful at the time of the assault and reported during the initial examination, excluding the pelvic area, and the body area that was painful 6 weeks and 3 months after the event, such as the cervical or abdominal area. These physical symptoms are significantly more severe and interfere more in the lives of survivors compared to the general population, e.g., the number of days spent in bed or with restricted activity is higher among survivors. A third study [32] shows lower satisfaction with physical health among survivors.

#### 3.2.3. Sexual Impairment

Four studies [23,24,25,31], using five different scales (Female Sexual Function Index, Larson Sexual Satisfaction Questionnaire, Sexual Performance Index Questionnaire, Amsterdam Hyperactive Pelvic Floor Scale-Women, Subjective Birth Experience Questionnaire) show impaired sexual health. Sexual satisfaction, functionality, and sexual performance are all affected. The sexual dysfunctions found are vulvodynia, dyspareunia, irritable bowel syndrome, and lubrication problems.

#### 3.2.4. Psychological Impairment

Three studies [26,27,28] looked at psychological functional impairment using the General Health Questionnaire (GHQ), the subsections of which are physical symptoms, social dysfunction, anxiety, and depression. It is the only scale found to qualify a global psychological state. Studies show a higher score for sexual assault survivors, reflecting a limitation of usual activities due to the psychological condition or the severity of the psychiatric disorder in general. The score may also be a good indicator of morbidity.

#### 3.2.5. Social and Occupational Impairment

Two studies [26,32] investigate the social functional impact: social satisfaction and functioning are decreased and the self-esteem score is below average. This result is reminiscent of the result of the GHQ subgroup on social dysfunction.

No scale was reported for occupational impact.

#### 3.2.6. Quality of Life Impairment

The two studies [29,30] measuring overall impact used the same scale, the Medical Outcomes Study Short Form, as SF-12 or SF-36, describing a decrease in quality of life. These scales take into account a physical component based on discomfort in everyday life and a psychological component, asking about the feelings and sensations experienced over the last four weeks.

### 3.3. Assessment of the Quality of the Studies

Overall, the studies included comply with the STROBE recommendations and use scales or measures validated or standardized for the outcome (Appendix B). The reporting of these studies is therefore satisfactory on average. A total of 6 of the 13 studies were based on samples of fewer than 100 people, which may limit the validity of the results, especially for studies including around 35 to 40 people [24,26]. For a direct measurement of functional impact, this sample size is not too problematic, but it excludes any reasonable possibility of adjustment for covariates or confounding factors, which also limits the scope of the results.

Nevertheless, in terms of the general validity and quality of the studies included, several have limitations: the biases are not discussed, the majority of the studies are retrospective, and only four take as an explicit criterion for inclusion the fact of being a victim of sexual violence with a prospective design. Any missing data are not mentioned or discussed. Only half of the studies discuss or suggest that their results can be generalized and, therefore, have sufficient external validity.

Four studies appear to be of better quality than the others and relate to the following dimensions: social impact and coping strategies [20]; physical impact, particularly in terms of painful symptoms [25]; impact on sexual functioning in women [23]; and finally psychological impact, via the GHQ scale [27].

## 4. Discussion

### 4.1. Overall Results

We conducted a systematic review of the literature according to the PRISMA guidelines, which ensured the quality of the review according to established standards. In addition, the many inclusion and exclusion criteria forced the two researchers and the reviewer to read the articles in detail to inform their decision of whether to include them or not, unlike in some other studies [33].

As a main result, 13 articles out of 1130 eligible articles were selected. The 13 articles included investigated physical (n = 2), sexual (n = 4), psychological (n = 3), and social (n = 2) dimensions, as well as overall quality of life (n = 2). There were no articles on occupational impact. All these articles highlighted the impact of sexual violence: first of all, the physical domain was affected, with an increasing incidence of disabling physical symptoms and the number of days of restricted activity. As regards the sexual domain, a lower level of sexual satisfaction was found after the assault than before and is explained by a lack of desire, orgasm, lubrication, and an increase in pain during the act. The psychological impact is equally marked, with overall mental health scores lower than those of the average population. Social impact and quality of life scores also show a negative effect.

### 4.2. A Very Limited Body of Literature

The identification of these thirteen studies indicates two opposing elements: on the one hand, certain researchers have wondered about the possible functional impairment and its quantification in sexual violence survivors, independently of the existence of one or more pathologies that would be associated with them. On the other hand, this interest remains very marginal, despite the fact that the prevalence of sexual violence in the world is significant.

Of these 13 studies, we find that the design and the level of quality can be judged as rather suitable for only 4 of them. In addition, these four studies relate to four different dimensions of functional impairment. All in all, this means that we only have one study of satisfactory quality and a priori validity for each dimension considered. At this stage, discussing in detail the quantified results of these studies makes little sense; it would be imperative above all to be able to replicate these studies in order to compare the results and test their robustness, and then possibly to refine the precision in terms of quantifying the impairment measured.

#### 4.2.1. Social Support and Avoidant Coping: A Short-Term Coping Strategy That Can Be Adapted but That Is Likely to Be Deleterious in the Medium and Long Term for Survivors

The study [20] is partly interested in the coping strategies mobilized and associated with positive changes in people’s daily lives and the use of avoidant coping following sexual violence, among 171 women recruited in 7 emergency services and monitored several times over an entire year. The level of recourse to avoidant coping but also to self-blame behavior was above average among this population and remained at a more or less constant level over the year of follow-up, with a downward trend at the end of follow-up.

While avoidance may initially be an effective and appropriate strategy, because it avoids a reactivation of the trauma, it is not a medium- or long-term solution. Depending on the characteristics of the violence, avoidance can have a major impact on everyday life. If the violence occurred in a place usually frequented by the survivor, or even a place through which she must pass frequently, having to avoid this place can complicate daily life to a greater or lesser extent. If the avoidance concerns sexual relations, regardless of the partner, this can also be a source of suffering. Moreover, self-blaming can impact self-esteem in the more or less long term and thus reinforce the impact of the consequences on the functioning of the assault survivors.

#### 4.2.2. Physical Impairment and Pain Evaluation: A Multisite and Lasting Pain in More Than One in Four Survivors

The study [21] is specifically interested in physical impairment, via the evaluation of the pain expressed and its anatomical location, over time (at 1 week, 6 weeks, and 3 months), in 84 women recruited during their use of care within 48 h of sexual assault. This study highlights how functional impairment can be expressed differentially, even within a single (physical) dimension, in terms of anatomical locations, number of anatomical sites affected, and persistence over time, but also that there may be significant functional repercussions independently of any pathological consideration. More specifically, pain was reported for one body region in 58% of cases at 6 weeks and 60% at 3 months, and for three or more body regions in 32% of cases at 6 weeks and 28% at 3 months. More than 10% reported pain in five or more body regions. The highest prevalence was observed for the following anatomical regions: head, neck, back, and abdomen.

The persistence of pain at 3 months in more than a quarter of survivors should draw our attention. On the one hand, consultation for persistent pain, without a well-identified cause initially, can be linked to undeclared sexual violence. It may be important for the clinician to keep this possibility in mind. On the other hand, depending on the intensity of the pain, it can be a gateway to drug addiction, which itself should be monitored.

#### 4.2.3. Sexual Functional Impairment

The study [23] focused on the evaluation of sexual functional impairment via the use of the Female Sexual Function Index (FSFI) scale, within a population of 73 women, reviewed at 6 months of a rape or attempted rape, in an emergency clinic for rape survivors. More specifically, 60% of respondents estimated their sexual function 6 months post-assault as impaired. The mean FSFI total score was lower post-assault compared to pre-assault (23 vs. 28.7, *p* < 0.001).

Sexual activity, although variable from person to person, can be an important activity of daily living. If it is disturbed in a lasting way, this can significantly modify a person’s life, their relationship with themselves and their body, and their relationship with others, in particular in the case of a person with one or more intimate partners. This impact should be considered with the previous results. It is possible to combine, and therefore potentiate, the impact on sexual life with self-blame, loss of self-esteem, or even the persistence of pain.

#### 4.2.4. Psychological Impairment: The Most Important and Generalized Impairment in Survivors

The study [27] assessed psychological impairment through the use of the GHQ, with a population of 294 women recruited from a forensic medicine center and reviewed one month later. A GHQ score greater than four indicates a significant functional impact. This score was reached in 89 to 93% of the women included. Again, this is indeed a significant functional impairment, assessed independently of any specific associated pathology.

This last result is important, significant, and concerning. Indeed, the psychological repercussions, in addition to other possible repercussions in a quarter to more than half of the survivors, are observed in almost all of the survivors. Here too, the existence or persistence of general psychological symptoms, without any obvious cause, should raise the possibility of sexual violence. That the psychological impact is the most frequent is also currently problematic from a legal point of view because, unlike visible traumatic injuries, psychological symptoms are rarely considered as proof in themselves of violence. Finally, the psychological impact can, of course, impact all aspects of everyday life, or even contribute to degrading them, through a lack of sleep, concentration difficulties, loss of desire, irritability, and so on.

These four studies of reasonable quality indicate that there may be a significant functional impairment of variable presentation depending on the people reporting sexual assaults, in the physical, psychological, sexual, and social dimensions, and this is independent of any specific or unique pathology.

### 4.3. Limitations and Strengths

There are several limitations to this review. First, the search equation was performed using both Mesh terms (terms indexed by the authors to their articles) and non-Mesh terms. Not all authors use the same definitions of sexual violence or impairment. We mitigated this by using the broadest possible search equation, refining this equation as we searched by adding or removing terms, and benefiting from a research group that has already worked on violence and related literature searches. In addition, we studied the content of the articles in detail. We also used only one database. From experience, the study of functioning and the use of validated scales, with the possible exception of psychological functioning, is preferably published in biomedical journals, the majority of which are indexed in Medline. PsycInfo could possibly have been added, but our institution has limited access to this database and to the journals more specifically linked to it. In addition, the majority of peer-reviewed scientific journals dealing with sexual violence from a biomedical or epidemiological point of view are also referenced in Medline.

Furthermore, the objective was not to focus on a single, precise dimension of functioning, so that a meta-analysis would also have been possible—we knew that there would not be enough studies to include, but to account for the paucity of research on this issue. The very small number of studies included and concerning several dimensions of functioning implies that no robust conclusion can be drawn at this stage. Thus, the objective was not to put forward robust and precisely quantified conclusions regarding functioning. The optimization of the design via the accumulation of queried databases has only a limited meaning and interest in the present case. The effort and energy required to query other databases would have been disproportionate to the expected results.

The second limitation is that we did not use the associated pathologies to investigate the impact of these. The study of pathologies cannot replace or be assimilated into the direct measurement of the impairment. There is too great a variety of pathologies following a sexual assault to be able to study the functional impact of each one. Indeed, a diagnosis does not account for the impairment experienced by the survivor; a correlation must exist between diagnosis and functional impairment, but it is likely to be very variable depending on the pathology and in general quite weak. The interindividual responses are very likely to be heterogeneous in terms of impact in relation to a given pathology. The functional impairment would have been better documented with the degree of severity, but the latter is not necessarily studied or reported.

A third limitation was that we only considered violence occurring at the age of at least 15 years and excluded polyvictimization and violence in childhood. This is both a limit and a strength of the study because it is what makes it possible to retain only results whose imputability in terms of functional impact on the experience of sexual violence is stronger. In the case of complex histories of violence dating back to childhood, or the accumulation of different types of violence, it becomes difficult to attribute such an impact to sexual violence only. Of course, future research should also look at these more complex cases.

Finally, the strengths of the study relate in particular to the fact of having complied with the PRISMA recommendations (Appendix A), with a detailed and blind screening of the articles identified, as well as having been able to report on the general quality of the studies included.

### 4.4. Implications for Future Research

Disclosing sexual violence outside the private sphere can be seen by survivors as not appropriate [34] and can lead to several difficulties or bad experiences [35,36]. The physician, the health professional, is one of the trusted figures to turn to [37,38]. This professional can be confronted with two non-exclusive possibilities: there is a pathology to be taken care of, and it is their usual work, independent of the fact that it is linked to violence, or there is no particular pathology, and they may appear less prepared to deal with this possibility. In both cases, it is the person as a whole that must be considered and not only the pathology, or an isolated type of impact (for example, physical or psychological), and it is necessary to be able to reason in terms of functioning and impact on the person’s daily life. Indeed, it is impossible to know what dimensions and in what time frame the person will present difficulties, and the main concern of a victim may be above all “to get better.” Knowledge of pathologies and their management is well established in general and continues to be the subject of updated research. On the other hand, we note here that little, if anything, is undertaken to characterize the functional impact, which is the first step necessary for the development of recommendations for good practices and coordinated and adapted care.

Several additional steps can be considered for future research. Firstly, the study highlights an unfortunate lack of research on measuring the functional impact of sexual violence, despite it being a crucial issue for the lives of the people concerned and a measure of concrete disability. More studies, focusing in particular on the different dimensions of this impact, are therefore necessary. This also has an implication in legal terms: if the criminal conviction of a perpetrator of sexual violence is based on the characterization of this violence, reparation and compensation for survivors are precisely assessed based on the impact of this violence on all dimensions of survivors’ lives and over time. Not having such studies, therefore, implies that the expert opinions currently given are not based on any valid scientific data. Second, we targeted adults only, with no polyvictimization or history of childhood sexual abuse. This, of course, only reflects part of the reality. It would, therefore, be appropriate to explore and compare these other profiles with those of people reporting only sexual violence after the age of 15 in terms of impact, and to try to determine how the accumulation or combination of types of violence affects the impact. At our level, our team developed the I-ADViSe study in an interdisciplinary manner, including people reporting sexual violence in the 28 days preceding a consultation in one of the seven French forensic medicine centers participating in the study, and for whom we measure, at five points in time over the course of a year, using standardized scales and quantitative questionnaires, as well as semi-structured interviews, the impact and the psychosocial and legal pathways [39].

## 5. Conclusions

The knowledge and evaluation of the specific or global functioning of people following a stressful or traumatic life event are complementary to the knowledge and evaluation of the pathologies associated with these events. Function and pathology can be linked, correlated, or independent. In the case of sexual violence, whose lifetime prevalence in the world is significant, and which is rarely disclosed, functional impairment is ultimately poorly known, and the little evidence we have collected in this review suggests that it is multidimensional, of variable presentation according to the individual and the temporality, and impacts all dimensions of functioning: physical, psychological, social, and sexual. It is important that further research is conducted in this area in the near future.

## Figures and Tables

**Table 1:** Characteristics of included studies.

Study	Aim	Design	Population	Type of Sexual Violence	Functional Impact	Measure(s)	Stat. Methods	Results
**S1** [20] Frazier P, Journal of Consulting and Clinical Psychology, 2004 DOI: 14756611	Assess correlates of early reports of positive life changes after sexual assault and individual trajectories of self-reported positive change over time	Cohort prospective	N = 171 women, between 16 and 52 years Recruited from 7 emergency rooms 1-year follow-up (2 weeks, 2 months, 6 months, 12 months)	Sexual violence	**SOCIAL IMPACT** Social support Approach and avoidant coping Religious coping Perceived control Positive life changes	*Social Support Scale—2 items Cronbach: 0.81* *Coping Strategies Inventory—CSI—Cronbach: 0.91 for approach coping and 0.84 for avoidant coping* *Religious coping scale—Cronbach: 0.94* *Three scales from the Rape Attribution Questionnaire:* *Behavioral self-blame—Cronbach: 0.87* *Control over recovery—Cronbach: 0.84* *Taking precaution—Cronbach: 0.83* *14/17 items from 17-item life change scale*	Hierarchical linear modeling—HLM	The trajectories of social support, approach coping, religious coping, control over the recovery process and taking precautions were all positively associated with positive-change trajectories, suggesting that increases in these variables were associated with increases in self-reported positive life changes over time
**S2** [21] Ulirsch JC, European Journal of Pain, 2013 DOI: 24019249	Assess the incidence and distribution of pain symptoms and other types of somatic symptoms experienced by women sexual assault survivors	Prospective observational study	N = 84 women, 18 years or older (M = 26 years) Recruited through medical care within 48 h of sexual assault	Sexual assault (vaginal, oral, or anal penetration) No repeated assault	**PHYSICAL IMPACT** Evolution of pain at 1 week, 6 weeks, and 3 months 21 somatic symptoms (headache, dizziness, nausea, noise sensitivity, concentration difficulties, taking longer to think…)	***Numeric rating scale**—0 to 10—in 8 body regions* ***Numeric rating scale***	Incidence rates CI 95% Bonferroni adjusted	Pain reported for: - 1 body region: 58% after 6 weeks and 60% after 3 months - 3 or more body regions: 32% after 6 weeks and 28% after 3 months - More than 10% reported pain in 5 or more body regions Highest prevalence regions: head, neck, back, and abdomen Association between location of trauma during SA and location of pain after 6 weeks and 3 months Somatic symptoms at 6 weeks: 38% showed a significant increase in severity Somatic symptoms at 3 months: 52% showed a significant increase in severity
**S3** [22] Golding JM, Research in Nursing & Health, 1996 DOI: 8552801	Hypothesized that sexual assault history is associated with limitations in physical functioning	Prospective observational study	N = 6024 respondents 516 reported a history of sexual assault 52% were female (M = 42 years)	Sexual assault with the sexual assault instrument (62 items)	**PHYSICAL IMPACT** Physical symptoms Physical functioning	*39 items from the somatization section of the Diagnostic Interview Schedule (DIS). Symptoms were scored as positive only if they were severe, which was defined as causing the respondent to use medication other than aspirin or interfering significantly with their life* *Number of days spent in bed because of illness* *Number of days of restricted activity*		Physical symptoms reported in SA vs. control: 67.9 vs. 41.7 (*p* < 0.001) Both bed days and restricted activity days were significantly more common among persons with a history of sexual assault than among those without Number of (SA vs. control): - Bed days 15.9 vs. 10.1 (*p* = 0.006) - Restricted activity days 18.9 vs. 11.7 (*p* = 0.002)
**S4** [23] Högbeck I, Journal of Sex & Marital Therapy, 2022 DOI: 34338163	Investigate the effect of sexual assault on sexual function in adult women six months post-assault, as well as the possible correlations between impaired sexual function and assault characteristics, psychiatric morbidity, and post-assault PTSD	Prospective observational study	N = 73 women (M = 24 years) Over 18 years Seen within one month of the index assault in the emergency clinic for rape survivors	Rape or attempted rape (penetration or not) Sexual assault age: M = 24 years No information about repeated violence	**SEXUAL IMPACT** Sexual function (last 4 weeks)	***FEMALE SEXUAL FUNCTION INDEX** (38 items measuring sexual function before and after the assault)* *26.55 is the optimal cutoff for differentiating women with and without sexual dysfunction* *Cronbach coefficient: 0.92 to 0.98*	Paired simple *t*-test Comparisons use Pearson’s chi-square test	60% estimated their sexual function at six months post-assault as impaired The mean FSFI total score was lower post-assault compared to pre-assault: 23 vs. 28.7 (*p* < 0.001)
**S5** [24] Tadayon M, Electronic Physician, 2018 DOI: 29881522	Determine the relationship between sexual performance and sexual satisfaction in those who have experienced violence, so that we can take steps to improve women’s sexual health	Case-control study	N = 35 women aged 15–45 years, married for at least a year, who have been sexually abused and referred to a forensic medical examination unit (M = 28 years) N = 70 women in control group (M = 29 years)	Sexually abused women referred to a forensic medical examination unit	**SEXUAL IMPACT** Sexual satisfaction Sexual performance	***Larson Sexual Satisfaction Questionnaire**—25 items, 5-option Likert scale* *Dissatisfaction (score under 50), low satisfaction (50–75), moderate satisfaction (75–100), high satisfaction (>100)* *Cronbach coefficient: >0.7 validated in couple population* ***Sexual Performance Index Questionnaire**—6 subscales including libido, sexual desire, lubrication, orgasm, satisfaction, and pain—19 questions—scores below 26 considered as impaired performance* *Cronbach coefficient: >0.7*	Chi-square test, *t*-test, Fisher’s exact test	Significant difference between the two groups (SA vs. control) in terms of sexual satisfaction: 71.4 vs. 99.44 (*p* < 0.001) Significant difference regarding sexual performance (SA vs. control): 17.10 vs. 26.37 (*p* < 0.001) Statistically significant difference in terms of sexual arousal (decreased in SA), orgasm (id), lubrication (id), pain (increased in SA), and sexual satisfaction (decreased in SA)
**S6** [25] Postma R, Journal of Sexual Medicine, 2013 DOI: 23679151	Investigate the effect of rape on sexual problems and on pelvic floor problems	Cross-sectional study	N = 89 women raped during adolescence (M = 20.9 years) N = 114 women in the control group (M = 20.8 years)	Rape includes both attempted and completed rape 20% revictimization	**SEXUAL IMPACT** Sexual functioning Pelvic floor functioning	***Female Sexual Function Index FSFI**—cutoff: 26.55* *Cronbach coefficient: 0.92 to 0.98* ***Amsterdam Hyperactive Pelvic Floor Scale-Women*** *11.00 is the optimal score for differentiating women with and without pelvic floor dysfunction* *Cronbach coefficient: 0.75 to 0.82*	Sample *t*-test Pearson’s chi-square tests	Prevalence of sexual dysfunction in SA vs. control: 44.6% (N = 25/56) vs. 19.5% (N = 15/77) Significant difference t: rape survivors had a 2.4 times higher prevalence of sexual dysfunction when compared with the control group More sexual problems for rape survivors: more lubrication problems (D = 0.497, *p* < 0.001) and more pain (D = 0.695, *p* < 0.001) Prevalence of pelvic pain in SA vs. control: 33.7% (N = 30/89) vs. 12.4% (N = 14/113) Significant difference: rape survivors had a 2.7 times higher prevalence of pelvic floor dysfunction More significant pelvic floor problems in SA: provoked vulvodynia (*p* < 0.001), stress (*p* < 0.001), lower urinary tract symptoms (*p* < 0.001), and irritable bowel syndrome symptoms (*p* < 0.001)
**S7** [26] Walker J, British Journal of Clinical Psychology, 2005 DOI: 10.1348	Investigate the effects of rape on a non-clinical male sample by comparing them on standard tests with a control group with no prior history of sexual assault	Case-control study	N = 40 male rape survivors (M = 34.2 years) Age of assault: 70% between 16 and 25 years Control group: 40 men with same characteristics	Sexual assault No precision	**PSYCHOLOGICAL IMPACT** General health **PSYCHOLOGICAL IMPACT** Trust in the future **SOCIAL IMPACT** Current self-esteem	***General Health Questionnaire (GHQ)**—28 items—4-point response scale* *Cronbach coefficient: 0.85* ***World Assumptions Scale**: People’s basic assumptions about the world in which they live.* *32 items—6-point response scale* *Cronbach coefficient: 0.40 to 0.83* ***State Self-Esteem Scale**—20 items—5-point response scale—Cronbach coefficient: 0.9*	Using *t*-tests *p* = 0.01	Significantly higher score for the survivors on each GHQ subscale: somatic symptoms: 2.96 *p* = 0.005 social dysfunction: 4.78 *p* = 0.001 anxiety/insomnia: 4.62 *p* = 0.001 depression: 7.06 *p* = 0.001 No significant difference Only self-worth is lower among the survivors (*p* = 0.001) Significantly lower self-esteem scores in the survivors group (58.95 vs. 74.05, *p* = 0.001) and for each subscale (performance, social appearance)
**S8** [27] Seyller M, Obstetrics & Gynecology, 2016 DOI: 10.1097	Compare the consequences of sexual assault based on the relationship between female survivors and their assailant	Prospective observational study	N = 294 women (M = 23 years) separated into 3 groups: G1: intimate partner: 106 (M = 29 years) G2: acquaintance: 100 (M = 20 years) G3: stranger: 88 (M = 21 years)	Sexual assault (vaginal, anal, or oral penetration, attempts, sexual touching)	**PSYCHOLOGICAL IMPACT** General health at 1 month after sexual assault	***General Health Questionnaire (GHQ)**—28 items—4-point response scale—cutoff point of 4/5* *Cronbach coefficient: 0.85*	Univariate analyses	Score > 4 points Group 1: 89% Group 2: 93% Group 3: 93% The rates of disturbance of survivors of intimate partner violence are similar to those of other survivors
**S9** [28] Darves-Bornoz JM, European Journal of Obstetrics & Gynecology and Reproductive Biology, 1997 DOI: 9550204	Determine whether the General Health Questionnaire, a simple psychological instrument, could be useful to non-specialists in screening for psychologically traumatized rape survivors	Prospective observational study	N = 285 subjects (M = 22.5 years), with 24 men and 261 women Rape survivors attending a consultation for survivors of psychological trauma, within the University Hospital of Tours	Rape (sexual penetration)	**PSYCHOLOGICAL IMPACT** General health—psychological	***General Health Questionnaire (GHQ)**—28 items—4-point response scale—cutoff point of 4/5* *Cronbach coefficient: 0.95*	SPSS SYSTAT software	Mean score: 11.7, independent of age, gender 72% had a GHQ score equal to or higher than the threshold set at 4
**S10** [29] Aquino NM, Revista Saúde Pública, 2009 DOI: 19967257	Estimate the prevalence of sexual violence history among pregnant women and its association with the self-perception of health status	Cross-sectional study	N = 179 pregnant women (M = 24 years)	Unconsented sexual contact (penetration or not) and acts of a sexual nature but without physical contact, such as voyeurism or exposure to pornography	**QUALITY OF LIFE** Assessment of health-related quality of life using the Medical Outcomes Study: Physical and mental functioning	***12-Item Short-Form Health Survey—SF-12***	A linear regression analysis of variance	Sexual violence survivors showed significantly lower mean physical (42.2) and mental (37.4) scores than women with no history of sexual violence (51, *p* < 0.001)
**S11** [30] Reyhan Dağ Karataş, Criminal Behaviour and Mental Health, 2019 DOI: 32307807	Determine factors associated with PTSD among women who had been sexually assaulted by comparing those with and without PTSD	Cross-sectional cohort study	N = 78 women approached at least 1 month after assault (M = 27.53 years) PTSD group: 41 (M = 28 years) Non-PTSD group: 19 (M = 24.9 years)	Rape: none, vaginal, vaginal and/or other Repeated assault: 39% in PTSD group, 16% in control group	**QUALITY OF LIFE**	***Medical Outcomes Study Short Form—36-item—SF-36*** *Assess both positive and negative aspects of health status* *No Cronbach coefficient*	The Mann–Whitney U test used for non-normal variables	The PTSD group differs from the group without PTSD with regard to the higher prevalence of other health problems, including a sense of poor general health, low vitality, impaired social function, impaired physical function, impaired physical role, and impaired emotional role
**S12** [31] Gottfried R, Midwifery, 2015 DOI: 26324214	Focuses on the inter-relationships between sexual abuse occurring across the lifespan, distressed female sexual function, and childbirth	Quantitative longitudinal study	N = 300 third term singleton pregnant women (M = 31 years) Sexual abuse: 21%	Sexual abuse: illegal act of forcing another person into any form of sexual activity or contact against their will	**SEXUAL IMPACT** Sexual dysfunction Subjective severity of respondents’ childbirth experience	***Female Sexual Distress Scale-Revised*** *13 statements on a 5-point scale—cutoff >10 to discriminate women with/without female sexual dysfunction* *Cronbach coefficient: 0.96* ***Subjective Birth Experience Questionnaire**—13 items on a 5-point scale* *Cronbach coefficient: 0.77*	Null hypothesis tests, chi-square test, significance, *p*-value, confidence interval, logistic regression	Women with a history of sexual assault showed approximately twice the prevalence of antenatal/postpartum FSD compared to those without such history Sexual abuse was not found to be related to a negatively experienced birth
**S13** [32] Holder N, Journal of Interpersonal Violence, 2022 DOI: 10.1177	Compare profiles of psychosocial outcomes for veteran and non-veteran participants who were exposed to childhood, adult, or military sexual assault	Data from the Comparative Health Assessment Interview Research Study	N = 935 non-veteran women and men who reported sexual assault N = 301 non-veteran women and men who reported sexual assault at 18 years or older N = 399 non-veteran women and men who reported childhood sexual assault	Sexual assault: penetration and touching	**PHYSICAL IMPACT** Health functioning Health satisfaction **SOCIAL IMPACT** Social functioning Social satisfaction	***Well-Being Inventory*** *Six items—score range 1 to 5 * *Cronbach coefficient: 0.72* *Three items—score range 1 to 5* *Cronbach coefficient: 0.75* *Three items—score range 1 to 5* *Cronbach coefficient: 0.84* *Four items—score range 1 to 5* *Cronbach coefficient: 0.80*	Z-score comparing between trauma groups	The adult sexual assault group reported worse outcomes than the child sexual assault group: Z-score is higher (so worse) in social satisfaction and functioning and health satisfaction. The score is more or less the same in health functioning WBI health functioning: 3.47 (0.69) WBI health satisfaction: 3.35 (1.06) WBI social functioning: 3.46 (0.99) WBI social satisfaction: 3.76 (0.93)

## Data Availability

Data sharing not applicable. No new data were created or analyzed in this study. Data sharing is not applicable to this article.

## Appendix A

**Table A1:** PRISMA checklist.

Section and Topic	Item #	Checklist Item	Location Where Item Is Reported
**TITLE**			
Title	1	Identify the report as a systematic review.	1
**ABSTRACT**			
Abstract	2	See the PRISMA 2020 for Abstracts checklist.	1
**INTRODUCTION**			
Rationale	3	Describe the rationale for the review in the context of existing knowledge.	1/2
Objectives	4	Provide an explicit statement of the objective(s) or question(s) the review addresses.	2
**METHODS**			
Eligibility criteria	5	Specify the inclusion and exclusion criteria for the review and how studies were grouped for the syntheses.	2
Information sources	6	Specify all databases, registers, websites, organisations, reference lists and other sources searched or consulted to identify studies. Specify the date when each source was last searched or consulted.	2
Search strategy	7	Present the full search strategies for all databases, registers and websites, including any filters and limits used.	2
Selection process	8	Specify the methods used to decide whether a study met the inclusion criteria of the review, including how many reviewers screened each record and each report retrieved, whether they worked independently, and if applicable, details of automation tools used in the process.	2/3
Data collection process	9	Specify the methods used to collect data from reports, including how many reviewers collected data from each report, whether they worked independently, any processes for obtaining or confirming data from study investigators, and if applicable, details of automation tools used in the process.	2/3
Data items	10a	List and define all outcomes for which data were sought. Specify whether all results that were compatible with each outcome domain in each study were sought (e.g., for all measures, time points, analyses), and if not, the methods used to decide which results to collect.	2/3
	10b	List and define all other variables for which data were sought (e.g., participant and intervention characteristics, funding sources). Describe any assumptions made about any missing or unclear information.	2
Study risk of bias assessment	11	Specify the methods used to assess risk of bias in the included studies, including details of the tool(s) used, how many reviewers assessed each study and whether they worked independently, and if applicable, details of automation tools used in the process.	3
Effect measures	12	Specify for each outcome the effect measure(s) (e.g., risk ratio, mean difference) used in the synthesis or presentation of results.	3
Synthesis methods	13a	Describe the processes used to decide which studies were eligible for each synthesis (e.g., tabulating the study intervention characteristics and comparing against the planned groups for each synthesis (item #5)).	3
	13b	Describe any methods required to prepare the data for presentation or synthesis, such as handling of missing summary statistics, or data conversions.	3
	13c	Describe any methods used to tabulate or visually display results of individual studies and syntheses.	3
	13d	Describe any methods used to synthesize results and provide a rationale for the choice(s). If meta-analysis was performed, describe the model(s), method(s) to identify the presence and extent of statistical heterogeneity, and software package(s) used.	3
	13e	Describe any methods used to explore possible causes of heterogeneity among study results (e.g., subgroup analysis, meta-regression).	3
	13f	Describe any sensitivity analyses conducted to assess robustness of the synthesized results.	3
Reporting bias assessment	14	Describe any methods used to assess risk of bias due to missing results in a synthesis (arising from reporting biases).	3
Certainty assessment	15	Describe any methods used to assess certainty (or confidence) in the body of evidence for an outcome.	3
**RESULTS**			
Study selection	16a	Describe the results of the search and selection process, from the number of records identified in the search to the number of studies included in the review, ideally using a flow diagram.	4
	16b	Cite studies that might appear to meet the inclusion criteria, but which were excluded, and explain why they were excluded.	4
Study characteristics	17	Cite each included study and present its characteristics.	4–12
Risk of bias in studies	18	Present assessments of risk of bias for each included study.	4–12
Results of individual studies	19	For all outcomes, present, for each study: (a) summary statistics for each group (where appropriate) and (b) an effect estimate and its precision (e.g., confidence/credible interval), ideally using structured tables or plots.	4–12
Results of syntheses	20a	For each synthesis, briefly summarise the characteristics and risk of bias among contributing studies.	4–12
	20b	Present results of all statistical syntheses conducted. If meta-analysis was done, present for each the summary estimate and its precision (e.g., confidence/credible interval) and measures of statistical heterogeneity. If comparing groups, describe the direction of the effect.	4–12
	20c	Present results of all investigations of possible causes of heterogeneity among study results.	4–12
	20d	Present results of all sensitivity analyses conducted to assess the robustness of the synthesized results.	4–12
Reporting biases	21	Present assessments of risk of bias due to missing results (arising from reporting biases) for each synthesis assessed.	4–12
Certainty of evidence	22	Present assessments of certainty (or confidence) in the body of evidence for each outcome assessed.	4–12
**DISCUSSION**			
Discussion	23a	Provide a general interpretation of the results in the context of other evidence.	13
	23b	Discuss any limitations of the evidence included in the review.	14
	23c	Discuss any limitations of the review processes used.	14
	23d	Discuss implications of the results for practice, policy, and future research.	15
**OTHER INFORMATION**			
Registration and protocol	24a	Provide registration information for the review, including register name and registration number, or state that the review was not registered.	
	24b	Indicate where the review protocol can be accessed, or state that a protocol was not prepared.	
	24c	Describe and explain any amendments to information provided at registration or in the protocol.	
Support	25	Describe sources of financial or non-financial support for the review, and the role of the funders or sponsors in the review.	
Competing interests	26	Declare any competing interests of review authors.	

## Appendix B

**Table A2:** STROBE compliance for all included studies in the systematic review.

	S1	S2	S3	S4	S6	S5	S7	S8	S9	S10	S11	S12	S13
Title and abstract	x	X	x	x	x	x	x	x	x	x	x	x	x
**Introduction**													
Background	x	x	x	x	x	x	x	x	x	x	x	x	x
Objectives	x	x	x	x	x	x	x	x	x	x	x	x	x
**Methods**													
Study design	x	x	x	x	x	x	x	x	x	x	x	x	x
Setting	x	x	x	x	x	x	x	x	x	x	x	x	x
Participants	x	x	x	x	x	x	x	x	x	x	x	x	x
Variables	x	x	x	x	x	x	x	x	x	x	x	x	x
Data sources	x	x	x	x	x	x	x	x	x	x	x	x	x
Bias													
Study size	x	x	x	x	x	x	x	x	x	x	x		x
Quantitative variables	x	x	x	x	x	x	x	x	x	x	x	x	x
Statistical methods	x	x	x	x	x	x	x	x	x	x	x	x	x
**Results**													
Participants													
a numbers	x	x	x	x	x	x	in method part	x		x	x	x	x
b reasons for non-participation		x			x			x					x
c flow diagram		x						x					
Descriptive data													
a characteristics	x	x	x	x	x	x	x	x	x	x	x	x	x
B number of participants with missing data				x									x
*c cohort study:* summarise follow-up	x												
Outcome data	x	x		x	x	x	x	x		x	x	x	x
Main results													
a unadjusted estimates	x	x	x	x	x	x	x	x	x	x	x	x	x
b report category boundaries	x	x	x	x	x	x	x	x		x	x		x
c consider translating estimates				x		x		x					
Other analyses	x	x	x	x	x	x		x	x		x		x
**Discussion**													
Key results	x	x	x	x	x	x	x	x	x	x	x	x	x
Limitations	x	x		x	x	x	x	x		x	x	x	x
Interpretation	x	x	x	x	x	x	x	x	x	x	x	x	x
Generalisability	x			x	x	x		x			x		x
**Other information**													
Funding					x							x	x
Design	Cohort study	Observational study			Cross-sectionnal study	Case-control study	Case-control study	Prospective observational study		Cross-sectionnal study		Longitudinal study	Prospective observational study
